# Effects of intracerebroventricularly administered opioid peptide antagonists on tissue glycogen levels in rats after exercise

**DOI:** 10.3906/sag-2011-323

**Published:** 2021-08-30

**Authors:** Ayşe Şebnem İLHAN, Şevin GÜNEY, Sibel DİNCER

**Affiliations:** 1 Department of Basic Medical Sciences, Gülhane Faculty of Dentistry, University of Health Sciences, Ankara Turkey; 2 Department of Physiology, Faculty of Medicine, Gazi University, Ankara Turkey

**Keywords:** Exercise, glycogen, endogenous opioid peptide, naloxone, naloxone

## Abstract

**Background/aim:**

Physical exercise is a state of physiological stress that requires adaptation of the organism to physical activity. Glycogen is an important and essential energy source for muscle contraction. Skeletal muscle and liver are two important glycogen stores, and the energy required to maintain exercise in rodents are provided by destruction of this glycogen depot. In this study, the effects of endogenous opioid peptide antagonism at the central nervous system level on tissue glycogen content after exhaustive exercise were investigated.

**Materials and methods:**

Rats had intracerebroventricularly (icv) received nonspecific opioid peptide receptor antagonist, naloxone (50 μg/10 μL in saline) and δ-opioid receptor-selective antagonist naltrindole (50 μg/10 μL in saline) and then exercised till exhaustion. After exhaustion, skeletal muscle, heart, and liver were excised immediately.

**Results:**

Both opioid peptide antagonists decreased glycogen levels in skeletal muscle. Although, in soleus muscle, this decrease was not statistically significant (p > 0.05), in gastrocnemius muscle, it was significant in the icv naloxone administered group compared with control (p < 0.05). Heart glycogen levels increased significantly in both naloxone and naltrindole groups compared to control and sham-operated groups (p < 0.05). Heart glycogen levels were higher in the naloxone group than naltrindole (p < 0.05). Liver glycogen levels were elevated significantly with icv naloxone administration compared with the control group (p < 0.05). Glycogen levels in the naloxone group was also significantly higher than the naltrindole group (p < 0.05).

**Conclusion:**

Our findings indicate that icv administered opioid peptide antagonists may play a role in glycogen metabolism in peripheral tissues such as skeletal muscle, heart, and liver.

## 1. Introduction

Physical exercise is a state of physiological stress that requires adaptation of the organism to physical activity. Exercise causes an increase in heart rate, blood pressure, tidal volume, and respiratory capacity but also affects the levels of various hormones and metabolites [1].

Glycogen is an important and essential energy source for muscle contraction. Skeletal muscle and liver are two important glycogen stores [2], and the energy required to maintain exercise in rodents is provided by destruction of this glycogen storage. Although muscle glycogen is the biggest source of carbohydrates during exercise, the ratio of destruction depends on the severity of exercise [3]. When the duration of an exercise at a certain severity is prolonged, a decrease in glycogen level occurs [4]. Various hormonal and central factors can affect the depletion of muscle glycogen depots during exercise [5,6,7]. Exercise is perceived by the organism as a stress factor [8]. It is known to increase the secretion of endogenous opioid peptides (EOPs) such as β-endorphins, as well as many stress hormones [9].

Endorphins are released from the anterior pituitary gland with adrenocorticotropic hormone (ACTH) in response to stress and in direct proportion to the severity of exercise [10,11]. EOPs are thought to play an important role in the regulation of hormonal and metabolic responses to exercise. It has been shown that increased serum β-endorphin concentration due to exercise can be associated with various psychological and physiological changes in, such as, mood, perception of pain, hormone levels like ACTH, growth hormone, prolactin, catecholamines, and cortisol, and menstrual irregularities in female athletes [12,13].

Endogenous opioid peptides, especially β-endorphins, have been shown to facilitate muscle glucose uptake during exercise [14]. Although there is no evidence that EOPs have a direct effect on glycogen metabolism, they can be expected to be effective on glycogen metabolism by seriously affecting glucose uptake. Endogenous opioid peptide receptors are known to be widely distributed in areas related to glucose homeostases, such as the pancreas and pituitary gland [15]. Although there are studies, which show that centrally administered morphine and similar compounds may affect glycogen levels in peripheral tissues such as liver [16], there is no study showing how central opioid peptide receptor antagonism affects glycogen levels in peripheral tissues in relation to exercise. 

As is known, physiological events and diseases that disrupt the regulation of central opioid release could also affect glycogen metabolism. Therefore, it is necessary to clarify the effects of central opioid peptides (OPs) on glycogen levels in peripheral tissues. Thus, in this study, we aimed to investigate the effects of intracerebroventricularly administered opioid peptide receptor antagonists on glycogen levels in peripheral tissues such as skeletal muscle, heart, and liver after exhausting exercise in rats.

## 2. Materials and methods

In this study, forty Wistar albino male rats with an average weight of 210 g were used. The rats were fed with standard chow and tap water ad libitum and lived at 12:12 hr light-dark cycle with room temperature at 21 °C as standard animal care condition. According to administrations, rats were divided into five groups as Control (C, n = 8), Sham (Sh, n = 8), Saline (S, n = 8), Naloxone (NX, n = 8), and Naltrindole (NT, n = 8). In all groups, rats had an exhausting exercise. Among the subjects, those who were injured or refused to run and those who couldn’t tolerate isoflurane sedation were excluded. This study was approved by Gazi University Ethical Committee for Experimental Research on Animals.

Preliminary studies were carried out for the subjects to familiarize the treadmill. All subjects were exercised on the treadmill for a total of 10 min/day for five consecutive days before performing stereotaxic surgery. In this period the speed was 7 m/min and 10 m/min in the first two days then progressively increased to 20 m/min, the angle was always 15° till the familiarization period.

Except for the control group, guide cannulas were placed in the left lateral cerebral ventricles of all animals for icv administrations. For this purpose, the subjects were anesthetized with alphazyn (10 mg/kg) and ketamine (90 mg/kg) mixture and then placed in a stereotaxic apparatus. The coordinates for icv application were determined as Anterioposterior (A): –0.8 mm, Lateral (L): –1.5 mm and, Dorsoventral (DV): –3.8 mm using the rat brain atlas [17] and referring to the junction point of the lambda and bregma point on the skull. The coordinates were also determined with preliminary experiments and confirmed by methylene blue (Figure) and histological sections.

**Figure:**
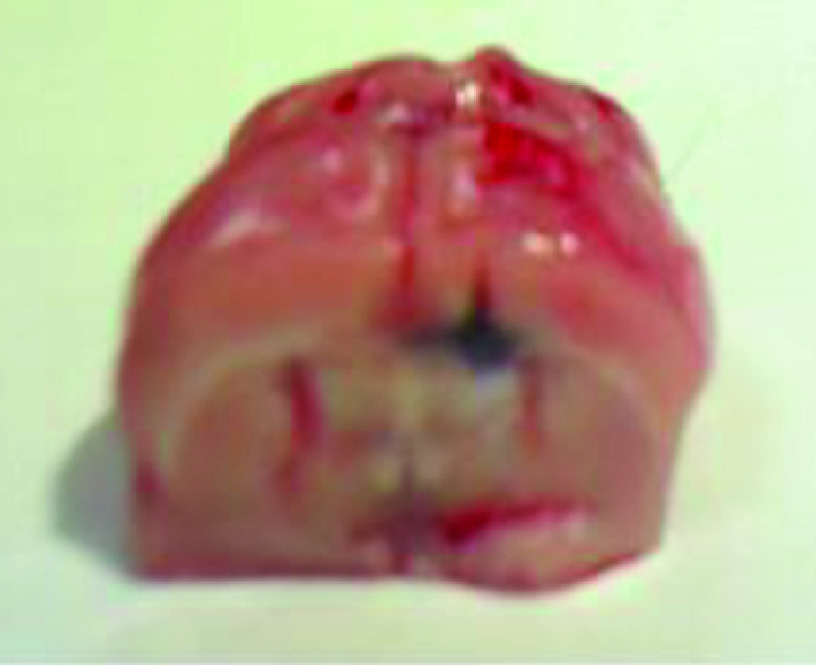
Verification of intracerebroventricular coordinates with methylene blue application.

Rats in all groups were sedated 90 min before the exhaustive exercise with isoflurane inhalation. Using a Hamilton injector, 10 μL saline, 50 μg / 10 μL naloxone (Sigma Aldrich, Germany) and 50 μg /10 μL naltrindole (Sigma Aldrich, Germany) were intracerebroventricularly administered to the subjects in saline, naloxone, and naltrindole groups, respectively.

Rats were subjected to exercise until they were exhausted on a treadmill at 20 m/ min speed and 15° incline. When the subjects refused to run and remained in the supine position for more than 10 s, they were determined as “exhausted”. Blood samples were collected from the heart of rats that were lightly sedated with isoflurane immediately after exhaustion. Rats were decapitated after cervical dislocation, and rapidly all tissues were excised and frozen in liquid nitrogen, stored in a –80 °C freezer for glycogen determination.

Tissue glycogen levels were measured according to the method of Lo S et al. [18]. Tissue samples weighing 0.3 gr. were placed in test tubes and then 1.5 mL 30% KOH (Merck) saturated with Na_2_SO_4_ (Merck) was added. The tubes were boiled in a boiling water bath for 30 min until a homogeneous solution was obtained, then cooled on ice. An equal volume (1.5 mL) of 96% ethanol with the KOH was added. Tissues were kept on ice for 30 min and then centrifuged at 2400 rpm for 30 min. Supernatants were discarded; the remaining pellets were dissolved in each of 3 mL distilled water. The sample in each tube was mixed with a glass rod for an equal time to dissolve the glycogen in the pellets. 1 mL of this mixture was taken and 1 mL of phenol (Fluka, Germany) and 5 mL of 96-98% H_2_SO_4_ (Merck) were added to them. 1 mL phenol and 5 mL 96%–98% H_2_SO_4_ were added on standard tubes containing 1 mL of distilled water and 5 mg/mL glycogen standard (Sigma Aldrich, Germany). Samples in all tubes were vortexed and incubated for 10 min at room temperature. After the incubation, the tubes were kept in a water bath set at 30 °C for 20 min. 300 μL samples taken from each tube were placed in a 96-well plate and were read spectrophotometrically at 490 nm against standard and blank. The absorbance of the samples was expressed as mg glycogen / g tissue.

### 2.1. Statistical analysis

Data analysis was performed using the Statistical Package for Social Sciences v.23.0 (IBM Corp., Armonk, NY, USA). Kruskal–Wallis test was used whether there was any difference between the treatments in terms of glycogen level in soleus, gastrocnemius, cardiac, and liver tissues. Bonferroni correction was used for the determination of where the difference originated. Whether there is a difference between administrations in each group, it was compared with Mann–Whitney U Test. Values were expressed as mean ± standard deviation (SD), standard error of the mean (SEM), and median (minimum, maximum), and p values <0.05 were considered as statistically significant. The effect size in calculating the sample size is 0.60, the type I error was taken as 0.05 and the power value as 0.80.

## 3. Results

### 3.1. Soleus muscle glycogen level

The soleus muscle glycogen levels of rats after exhausting exercise are shown in Table 1 as mean ± SD and SEM, also, in Table 2, as median (min, max). When the soleus muscle glycogen levels were compared in terms of administrations, no statistically significant difference was observed between the groups (p > 0.05).

**Table 1:** Mean, standard deviation, and standard error of the mean values of glycogen levels in soleus, gastrocnemius, cardiac, and liver tissue samples of rats after exhaustive exercise.

Gr	Soleus		Gastrocnemius		Cardiac		Liver	
	Mean ± SD	SEM	Mean ± SD	SEM	Mean ± SD	SEM	Mean ± SD	SEM
C	0.70 ± 0.52	0.23	1.16 ± 0.53	0.24	1.36 ± 0.11	0.05	1.88 ± 0.51	0.23
SH	1.43 ± 2.29	1.02	1.00 ± 1.07	0.48	1.36 ± 0.20	0.09	2.21 ± 0.25	0.11
S	0.88 ± 1.92	0.68	0.70 ± 0.38	0.14	2.47 ± 1.00	0.35	2.27 ± 0.44	0.16
NX	0.4 ± 0.44	0.18	0.60 ± 0.62	0.25	3.21 ± 0.83	0.34	2.45 ± 0.38	0.15
NT	0.91 ± 1.11	0.39	0.83 ± 0.47	0.17	2.36 ± 0.16	0.05	1.96 ± 0.28	0.10

**Table 2:** Median (minimum, maximum) values of glycogen levels in soleus, gastrocnemius, cardiac and liver tissue samples of rats after exhaustive exercise.

Groups	Soleus median (min, max)	Gastrocnemius median (min, max)	Cardiac median (min, max)	Liver median (min, max)
C	0.90(0.02, 1.29)	1.06(0.60, 2.03)a	1.42(1.23, 1.47)a,b	1.73(1.31, 2.68)a
SH	0.82(0.01, 5.46)	0.70(0.27, 2.88)	1.29(1.11, 1.61)c,d	2.14(1.94, 2.56)
S	0.11(0.01, 5.60)	0.62(0.28, 1.38)	2.88(1.17, 3.62)	2.21(1.59, 2.90)
NX	0.34(0.02, 1.15)	0.44(0.14, 1.81)a	2.90(2.72, 4.88)a,c,e	2.41(2.04, 2.93)a,b
NT	0.44(0.03, 3.27)	0.90(0.26, 1.51)	2.32(2.17, 2.59)b,d,e	1.88(1.57, 2.54)b
p, Kruskal Wallis Test	> 0.05	< 0.05	< 0.05	< 0.05
p, Bonferroni correction		a C-NX= 0.045	a C-NX=0.006b C-NT=0.003c SH-NX=0.006d SH-NT=0.003e NX-NT=0.002	a C-NX=0.045b NX-NT=0.028

C: Control; SH: Sham; S: Saline; NX: Naloxone; NT: Naltrindole

### 3.2. Gastrocnemius muscle glycogen level

The gastrocnemius muscle glycogen levels of the rats after exhausting exercise are shown in Table 1 as mean ± SD and SEM, also, in Table 2, as median (min, max). When the gastrocnemius muscle glycogen levels were compared, in naloxone group, there was a statistically significant decrease compared to the control (p = 0.045).

### 3.3.Cardiac tissue glycogen level

The heart tissue glycogen levels of the rats after exhausting exercise are shown in Table 1 as mean ± SD and SEM, also, in Table 2, as median (min, max). When the groups were compared in terms of cardiac glycogen levels, there were significant increases in naloxone and naltrindole groups compared to control and sham groups (p = 0.006 and p = 0.003, respectively). When naloxone and naltrindole treated groups were compared, cardiac tissue glycogen levels in naloxone treated group was found to be statistically significantly higher than naltrindole treated group (p = 0.002).

### 3.4. Liver glycogen level

The liver tissue glycogen levels of the rats after exhausting exercise are shown in Table 1 as mean ± SD and SEM, also, in Table 2, as median (min, max). The glycogen level was found to be statistically significantly higher in the naloxone group compared to the control group (p = 0.045). When the groups treated with naloxone and naltrindole were compared, liver tissue glycogen levels were found to be statistically significantly higher in the group treated with naloxone compared to the group treated with naltrindole (p = 0.028). 

## 4. Discussion

In our study, we aimed to investigate the effects of central opioid peptide receptor antagonism on skeletal muscles such as soleus and gastrocnemius as well as heart and liver tissues after exhaustive exercise.

Opioid peptides are known to play a role in controlling blood glucose levels during rest and exercise. Circulating levels of β-endorphins, an important component of the opioid system, have been shown to increase with sufficient intensity and time of exercise [19]. There are many studies in the literature showing that β-endorphins and opioid peptide antagonists can be applied by different methods to investigate the role of endogenous opioid peptides in glucose homeostasis during exercise. Hashiguchi et al. administered intracerebroventricularly to demonstrate the effect of central opioids on glycogen levels in peripheral tissues as we did in the current study [16]. Radosevich et al. practiced on dogs to demonstrate icv and iv effects of β-endorphins on blood glucose level; they reported that central administration had increased blood glucose levels, whereas peripheral application did not affect glucose homeostasis [20]. Various studies also report that these effects of endogenous opioid peptides can be antagonized with naloxone in experimental conditions [21]. Angelopoulos et al. demonstrated that administration of iv naloxone antagonizes the glucagon response increasing the effect of endogenous opioid peptides during exercise in humans [22]. In literature, studies conducted to investigate the central effects of opioid peptides report that applications are generally performed as icv, and, for this purpose, guide cannulas are placed with the stereotaxic method after the selection of suitable coordinates. In our study, a guide cannula was placed in the left lateral cerebral ventricle with a similar method. We waited for a week in accordance with the literature [16,23] for the recovery after stereotaxic surgery. At the end of this period, glycogen levels in skeletal muscles, cardiac and liver tissues of the exhausted rats were investigated. Glycogen is the storage form of glucose found in most cell types and is an important and essential energy source for muscle contraction. Skeletal muscle and liver are the main important glycogen stores in the body as is known [2].

In rodents, both liver and muscle glycogen is largely used to maintain exercise. Skeletal muscles primarily use intramuscular glycogen depots during exercise [24], as is known.

Although muscle glycogen is the biggest source of carbohydrates during exercise; the rate of destruction depends on the severity of exercise [3]. Muscle glucose uptake during exercise is also correlated with the duration and severity of the exercise [25–27]. EOPs, especially β-endorphins, were shown to facilitate muscle glucose uptake during exercise [14]. Morphine is also known to be a hormone-like effect that facilitates glucose uptake by muscles. Morphine affects glucose uptake seriously and also affects glycogen metabolism [28]. Physical activity stimulates the release of β-endorphins into the circulation [29]. A strong, nonspecific opioid antagonist naloxone is mostly used in studies to explain the mechanisms related to β-endorphin secretion during exercise.

Both of the antagonists we used in our study reduced glycogen levels in skeletal muscles. Although decreases in glycogen levels are not significant in soleus muscle, in the naloxone group, glycogen levels of the gastrocnemius muscle were found to be significantly decreased compared to the control group. This difference may be due to the different types of muscle fibers.

As is known, soleus muscle is slower, and gastrocnemius muscle has more fast-twitch muscle fibers. Evans et al. have shown that the concentration of β-endorphin receptors is higher in fast muscles; hence, stimulation of muscle glucose uptake through β-endorphins in fast muscles is greater [30]. Probably, this is the reason of why glycogen levels in the gastrocnemius muscle are generally higher than the soleus muscle in all groups.

A significant decrease of glycogen levels in naloxone group of gastrocnemius muscle suggests that the effects of β-endorphins to increase muscle glucose uptake peripherally may be prevented by central antagonism. Therefore, we think that present glycogen stores are spent to supply the energy needs of the muscle. It is known that endorphins-containing nerve cells and opioid peptide receptors are widely distributed in nuclei of the central nervous system, which play a role in the autonomic regulation of the cardiovascular system [31,32]. Otherwise, the presence of opioid receptors on autonomic targets such as the cardiovascular system or lungs proves the presence of direct effects of endorphins in these tissues [33].

Myocardial cells are known to have the ability to synthesize, store and secrete opioid peptides [34]. Three types of opioid peptides (encephalins, endorphins, and dynorphins) are synthesized in the heart, and the presence of preproenkephalin, prodynorphin, and proopiomelanocortin precursor gene expressions in atrial [35] and ventricular [35,36] cardiac myocytes are reported in the literature.

In our study, cardiac glycogen levels in all groups were found higher than skeletal muscle levels. This may be related to the energy metabolism of the heart being different from skeletal muscles. The energy required for myocardial function during rest and exercise is provided by glucose, fatty acids, and lactate. While all three substrates contribute to the reconstruction of ATP at rest, most of the energy comes from free fatty acid breakdown. In the above-moderate exercise, lactate influx to the blood from active skeletal muscle increases a lot, and the heart provides most of its energy by oxidation of circulating lactate. In prolonged submaximal exercise, the use of free fatty acids in myocardial metabolism becomes more prominent in supplying the total energy need [37]. This can be the reason why cardiac tissue glycogen level is higher than skeletal muscles in all groups. During exercise, the energy need of the heart is likely to be obtained from other sources, and, therefore, the use of glycogen is relatively low. In our previous study, we obtained that blood lactate levels after exhausting exercise were higher than pre-exercise levels in all groups [38]. Cardiac glycogen can be higher than skeletal muscle levels because of the heart’s using circulating lactate during exhausting exercise as an energy source. When the cardiac tissue is compared in terms of administrations, it has been observed that the opioid peptide antagonism increases the glycogen content of the heart. This increase was found to be significant compared to both control and sham groups; nevertheless, the absence of a significant difference between control and sham groups supports that this effect is caused by antagonist administration.

In our study, post-exercise liver glycogen levels were also studied. Glycogen levels in naloxone group were higher among all groups. This increase was statistically significant when compared with the control and naltrindole groups. Hashiguchi et al. showed that intravenously administered morphine and its active metabolites do not alter glycogen levels in peripheral tissues of rats, while intracerebroventricular administered morphine reduces liver glycogen content [16]. Researchers stated that the cannula insertion process for icv administration led to an acute stress response and caused significant increases in plasma corticosterone and catecholamine levels [39]. Akil et al. also demonstrated a parallel increase in plasma adrenocorticotropic hormone (ACTH) and β-endorphine levels in case of stress [15]. Gunion et al. suggested that the roles of opioids in stress response are achieved by stimulating the pituitary-adrenal axis and μ-receptors that activate the sympathetic nervous system [40]. It was shown that endorphins play a role in neurotransmission in sympathetic ganglia in central nervous system and plexuses of the digestive system; in the periphery, it was also shown that they are released into circulation to play a possible hormone role by stimulation of pituitary gland and adrenal medulla [33]. Many studies also support that catecholamines and corticosterone can modulate the liver glycogen content. It is reported that epinephrine induces glycogenolysis by modulating phosphorylase activity in the liver and skeletal muscle; corticosterone increases glycogenolytic effects of epinephrine. Thus, hormonal changes caused by icv opiates cause a decrease in liver glycogen levels [16]. Since the organism perceives exercise as a stress factor [8], plasma cortisol and catecholamine levels increase significantly during moderate and high-intensity exercise [41,42].

Our findings have shown that intracerebroventricular opioid antagonism causes an increase in liver glycogen levels. This increase is more significant, especially in the naloxone group. Naloxone is known to be a nonspecific antagonist and has a greater affinity for the μ-receptor, although it binds to all three opioid receptors.

In our study, although hormone levels such as cortisol and catecholamine were not examined; we think that activation of pituitary-adrenal axis and possible catecholamine release induced by opioid peptides during exercise may be prevented by centrally administered opioid peptide antagonists. Thus, the glycogenolytic effects of endogenous opioid peptides on the liver via the pituitary-adrenal axis and sympathetic nervous system may have decreased relatively, and, therefore, liver glycogen levels may remain higher in the naloxone-treated group.

As a result, our findings show that opioid peptide antagonists administered intracerebroventricularly may have an effect on glycogen levels in peripheral tissues such as skeletal muscle, heart, and liver. Although there are many studies in the literature on central opioid peptide antagonism and exhausting exercise, no other studies have been found on how central antagonism affects glycogen levels in peripheral tissues after exhausting exercise. On the other hand, since NX and NT are also inverse agonists [43,44], the basal activity of endogenous OPR has decreased, thus, allowing us to see the effects of OPR on glycogen levels more clearly. However, in order to explain the existing and possible mechanisms in more detail, further studies are needed.

## References

[ref1] (1989). Energy Sources In: The physiological basis of physical education and athletics. Dubuque (Iowa): wcb Publishers.

[ref2] (2001). Regulation of glycogen metabolism. (NY).

[ref3] (1985). Effect of varying exercise intensity on glycogen depletion in human muscle fibers. Acta Physiologica Scandinavica.

[ref4] (1985). Influence of muscle glycogen on glycogenolysis and glucose uptake during exercise. Journal of Applied Physiology.

[ref5] (2003). Exercise starts and ends in the brai. European Journal of Applied Physiology.

[ref6] (2001). Evidence that a central governor regulates exercise performance during acute hypoxia and hyperoxia. The Journal of Experimental Biology.

[ref7] (1994). Metabolic effects of opiates and opioid peptides. Advances in Neuroimmunology.

[ref8] (2009). Exhaustive exercise causes an anti-inflammatory effect in skeletal muscle and a pro-inflammatory effect in adipose tissue in rats. European Journal of Applied Physiology.

[ref9] (1977). β-endorphin and adrenocorticotropin are selected concomitantly by the pituitary gland. Science.

[ref10] (1990). The influence of fitness on neuroendocrine responses to exhaustive treadmill exercise. European Journal of Applied Physiology and Occupational Physiology.

[ref11] (1989). Opioids and exercise. An update. Sports Medicine.

[ref12] (1984). Endorphins and exercise. Sports Medicine.

[ref13] (2001). Endogenous opiates: 2000. Peptides.

[ref14] (1997). Insulin and glucagon immunoreactivity during high-intensity exercise under opiate blockade. European Journal of Applied Physiology and Occupational Physiology.

[ref15] (1984). Endogenous opioids: biology and function. Annual Review of Neuroscience.

[ref16] (1998). Differential responses of brain, liver, and muscle glycogen to opiates and surgical stress. Surgery Today.

[ref17] (1998). The rat brain: in stereotaxic coordinates.

[ref18] (1970). Determination of glycogen in small tissue samples. Journal of Applied Physiology.

[ref19] (1997). Beta-endorphin response to exercise. An update. Sports Medicine.

[ref20] (1989). Central effects of beta-endorphins on glucose homeostasis in the conscious dog. The American Journal of Physiology.

[ref21] (2011). Porreca F et al. Anesthesiology.

[ref22] (1997). Insulin and glucagon immunoreactivity during high-intensity exercise under opiate blockade. European Journal of Applied Physiology and Occupational Physiology.

[ref23] (2003). Evidence that tryptophan reduces mechanical efficiency and running performance in rats. Pharmacology, Biochemistry, and Behavior.

[ref24] (1999). Postexercise glucose uptake and glycogen synthesis in skeletal muscle from GLUT4-deficient mice. FASEB journal: Official Publication of the Federation of American Societies for Experimental Biology.

[ref25] (1993). Regulation of endogenous fat and carbohydrate metabolism in relation to exercise intensity and duration. The American Journal of Physiology.

[ref26] (1986). Leg glucose uptake during maximal dynamic exercise in humans. The American Journal of Physiology.

[ref27] (1991). Regulation of glucose utilization in human skeletal muscle during moderate dynamic exercise. The American Journal of Physiology.

[ref28] (1963). Effects of morphine on the hormonal control of metabolism. I. In vitro effects of morphine and hydrocortisone on utilization of glucose by muscle of normal and chronically morphinised rats. Biochemical Pharmacology.

[ref29] (1992). Changes in beta-endorphin levels in response to aerobic and anaerobic exercise. Sports Medicine.

[ref30] (2001). Delta opioid receptors mediate glucose uptake in skeletal muscles of lean and obese-diabetic (ob/ob) mice. Metabolism.

[ref31] (1976). Immunohistochemical studies using antibodies to leucine-enkephalin: initial observations on the nervous system of the rat. Neuroscience.

[ref32] (1980). Differentiation of delta and mu opiate receptor localizations by light microscopic autoradiography. Proceedings of the National Academy of Sciences of the United States of America.

[ref33] (1983). Cardiovascular consequences of endogenous opiate antagonism. Biochemical Pharmacology.

[ref34] (1995). Pericardial repair depresses canine cardiac catecholamines and met-enkephalin. Regulatory Peptides.

[ref35] (1998). Opioid gene expression in the developing and adult rat heart. Developmental Dynamics.

[ref36] (1989). Preproenkephalin mRNA expression in developing rat heart and in cultured ventricular cardiac muscle cells. The Biochemical Journal.

[ref37] (2007). Exercise Physiology; Energy, Nutrition.

[ref38] (2012). The effects of intracerebroventrically administered opioid peptide receptor antagonists on exercise performance. Isokinetics and Exercise Science.

[ref39] (1995). -glucuronide: hyperglycemic and neuroendocrine potentiating effects. Brain Research.

[ref40] (1991). μ-receptor mediates elevated glucose and corticosteron after third ventricle injection of opioid peptides. The American Journal of Physiology.

[ref41] (2008). Exercise and circulating cortisol levels: the intensity threshold effect. Journal of Endocrinological Investigation.

[ref42] (2002). Effect of naloxone on perceived exertion and exercise capacity during maximal cycle ergometry. Journal of Applied Physiology.

[ref43] (2016). Constitutive desensitization of opioid receptors in peripheral sensory neurons. The Journal of Pharmacology and Experimental Therapeutics.

[ref44] (2019). Development of novel δ opioid receptor inverse agonists without a basic nitrogen atom and their antitussive effects in mice. ACS Chemical Neuroscience.

